# Creating an adolescent intensive health behavior and lifestyle treatment for type 1 diabetes: Feedback from the target population

**DOI:** 10.1016/j.obpill.2025.100194

**Published:** 2025-07-03

**Authors:** Jennifer L. Warnick, Katherine E. Darling, Smriti Maskey, Lisa Swartz Topor, Katelyn Fox, KayLoni Olson, Elissa Jelalian

**Affiliations:** a Department of Psychiatry and Human Behavior, Warren Alpert Medical School of Brown University, Providence, RI, USA; b Weight Control and Diabetes Research Center, The Miriam Hospital, Providence, RI, USA; c Department of Pediatrics, Warren Alpert Medical School of Brown University, Providence, RI, USA

**Keywords:** Adolescents, Obesity, Overweight, Type 1 diabetes

## Abstract

**Background:**

The prevalence of obesity among youth with type 1 diabetes (T1D) has significantly increased over the past few decades, leading to increased risks for morbidity and early mortality. Despite this growing need, there is a dearth of evidence-based intensive health behavior and lifestyle treatments (IHBLT) for youth with T1D. This study collected qualitative data from adolescents with T1D and with comorbid overweight/obesity and their caregivers to inform the refinement of an IHBLT specific to adolescents with T1D.

**Methods:**

This is an original clinical research study. Participants completed individual semi-structured interviews to provide feedback on planned session topics, handouts, and activities for a modified IHBLT for T1D. A deductive framework matrix analysis approach was used to analyze data. All data were analyzed by two independent coders who met to discuss divergence in coding and came to 100 % agreement. Codes were iteratively reduced and organized into themes.

**Results:**

Sixteen participants (*n* = 8 adolescents, *n* = 8 caregivers) completed qualitative interviews. Three main themes emerged from the data: (1) satisfaction with the proposed program, (2) program structure preferences, and (3) suggested adaptations.

**Conclusions:**

The adapted IHBLT for adolescents with T1D seems to be initially acceptable to the target population. Results provide suggestions for the continued refinement of an IHBLT specific to adolescents with T1D.

## Graphical abstract

**Figure:**
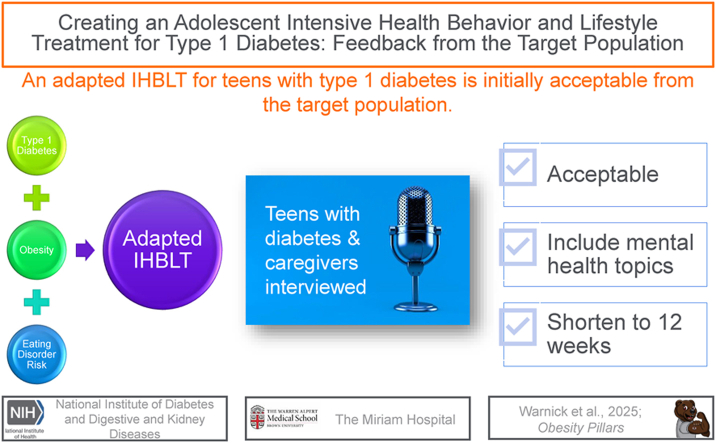

## 1 Introduction

The prevalence of obesity among youth with type 1 diabetes (T1D) exceeds that of youth in the general population [1] Youth with obesity are at increased risk for developing negative cardiometabolic health outcomes, which are leading causes of death and disability [2]. Regarding an emerging area of concern, youth with both obesity and with T1D may be at risk of developing insulin resistance to their exogenous insulin, increasing their risk for macro- and micro-vascular complications and additional weight gain [3,4]. Recent data indicate that over 50 % of youth with T1D will have overweight or obesity within 5 years of T1D diagnosis [5] As youth are commonly diagnosed with T1D between the ages of 10–14 years [6], adolescence is the optimal time to provide early intervention for obesity. Endocrinologists often prioritize diabetes management over weight management during routine appointments [7]. As a result, youth with T1D may not receive guidance around healthy weight management.

Intensive health behavior and lifestyle treatment (IHBLT) is the recommended first-line intervention for pediatric obesity [8]. Evidence-based IHBLTs involve at least 26 h of face-to-face treatment and provide psychoeducation and behavioral strategies emphasizing the following core concepts: balanced nutrition, individualized caloric deficit goal, increased physical activity, and family support to reduce excess adiposity [8,9]. Experts have specifically suggested lifestyle behavior change (e.g., increased physical activity and improved diet quality) for the treatment of obesity among youth with T1D [10]. However, previous studies indicate that factors involved in T1D management, such as managing hypoglycemia, low-carbohydrate diets, and a focus on disease management vs overall lifestyle, can be a barrier to some IHBLT recommendations [7,11]. Physical activity, without adjustments to dietary intake or insulin management, can lead to hypoglycemia, requiring a break in activity [12]. Managing hypoglycemia requires the consumption of fast-acting carbohydrates with low fiber and other nutrients (i.e., foods IHBLTs aim to limit). Treatment of one hypoglycemic episode per day can result in a 6-pound weight gain over a year [13]. Thus, an adolescent IHBLT curated specifically for T1D is needed.

Above and beyond these barriers, youth with T1D are at higher risk of developing disordered eating behaviors compared to youth without T1D [[14], [15], [16]]. Purposeful insulin omission to induce glycosuria, also known as “diabulimia,” is a dangerous behavior used by an estimated 20–40 % of youth with T1D for weight loss [14,16]. Though determining the exact prevalence of purposeful insulin omission for weight loss is difficult due to the self-report nature of assessment, being between 13 and 17 years old, having a diagnosis of overweight or obesity, and engaging in calorie counting are reported to be the strongest known associations for eating disorders in a T1D population according to published literature [15]. While there is consistent evidence that structured and professionally run IHBLTs reduce eating disorder prevalence, risk, and symptoms [17,18], any weight-related intervention specific to youth with T1D must carefully assess for disordered eating and include preventive strategies. Along the same lines, an IHBLT should be trauma-informed and include a non-stigmatizing approach when discussing disordered eating in this population.

To date, a T1D-specific IHBLT does not exist. Our team has previously interviewed adolescents with comorbid T1D and with overweight/obesity, their caregivers, and pediatric endocrinologists to gauge interest in a potential IHBLT specific to T1D [19]. Findings indicated high interest in an IHBLT specific to T1D, and participants indicated wanting education and skills related to diabetes-specific mental health problems. Participants provided mixed opinions regarding discussing weight directly. Most endocrinologists and caregivers expressed hesitation about discussing weight with teens, while most teens were interested in discussing weight loss. Participants made clear that meeting other adolescents and families with T1D would be a major draw to the program. Based upon this formative work, an evidence-based adolescent IHBLT [20] was adapted for adolescents with T1D. The current study sought specific feedback on this adapted adolescent IHBLT for T1D to ensure optimal acceptability and feasibility [21].

## 2 Materials & methods

### 2.1 Participants

Adolescents were eligible if they were: between the ages of 13–17 years old with a diagnosis of T1D for at least six months, had a body mass index (BMI) ≥ 85th percentile for gender and age, and spoke fluent English. Adolescent participants also required one English-speaking legal guardian to agree to participate in the study. Participants were recruited throughout the local diabetes community. Interested caregivers completed an online consent to contact form via REDCap hosted at Brown University Health [22]. Study staff then completed phone screens to verify eligibility and scheduled informed consent and interview appointments.

Forty-one caregivers completed the consent to contact form online expressing interest in the study. Eleven adolescent-caregiver dyads were eligible from the phone screen and scheduled study appointments. Of the remaining 30 dyads, seven dyads were not interested in the study, four teens did not meet the BMI inclusion criteria, and the remaining dyads could not be reached by their self-reported contact information. Eight dyads completed the study; three dyads cancelled or no-showed their study appointments.

### 2.2 Procedures

All study procedures were approved by the Brown University Health Institutional Review Board (IRB#: 1842958). Participants' legal guardians completed the consent process online via REDCap and Brown University Health's HIPAA-compliant Zoom. Adolescent participants completed an assent using the same methods. After consent/assent, participants each completed an individual semi-structured interview via Zoom or in person where they were asked to provide feedback on the planned IHBLT program session topics, handouts, and activities. Following the interviews, participants completed a demographics survey. Separate interview guides were used for adolescents and caregivers, who were interviewed independently. Interview guides were parallel except for caregivers being asked more directly about the content proposed for the caregiver sessions within the planned program, while adolescents were asked more detailed questions about planned session topics and handouts that only the adolescent participants would complete. Semi-structured questions allowed the interviewer to ask follow-up questions to clarify responses and gather additional detail and explanation. Interviews lasted approximately 1 h. Sample size was based upon the specificity of the research questions and purposeful sampling [23].

### 2.3 Data analysis

Each interview was audio-recorded, transcribed verbatim, and de-identified by the study team. A deductive framework matrix analysis was used for data analysis [24]. Two PhD-level independent coders with qualitative experience familiarized themselves with the interview data and created a matrix framework based on the interview questions, specific treatment components on which participants provided feedback, and patterns within the data. The coders went through a process of matrix refinement by independently coding four transcripts, discussing and refining the code structure until they came to full agreement on the final matrix design. Both coders coded all interviews. Coders met regularly to clarify discrepancies and came to 100 % agreement on all final codes. Following this process, the two coders identified emergent themes and iteratively reduced the data to identify final themes and demonstrative participant quotes. JW collected and analyzed the data; KED analyzed the data. Both are doctorate-level pediatric psychologists and research scientists. Neither works clinically with participants enrolled in this study. They work on diabetes-specific clinical research projects and volunteer with diabetes communities locally and nationally.

## 3 Results

### 3.1 Overview

Participants included eight adolescents with T1D and their caregivers. Demographic information is in Table 1. Three main themes, with additional subthemes, were identified. These themes are: (1) Satisfaction with the Proposed Program; (2) Program Structure Preferences; and (3) Suggested Adaptations.

**Table 1:** Demographic characteristics.

Characteristics	N (or *Mean*)	% (or *SD*)	Range
***Adolescent Characteristics (n = 8)***			
Gender identity			
Female	5	62.5 %	
Male	3	37.5 %	
Age(yrs)	*M =* 14.88	*SD =* 1.45	13–17
Race			
Multiracial	2	25.0 %	
White	5	62.5 %	
Other	1	12.5 %	
BMI percentile for age and gender	*M =* 93.83	*SD =4*.39	88–99.6
Has overweight	4	50 %	
Has obesity	4	50 %	
Years with T1D	*M* = 5.94	*SD* = 4.09	0.5–13.0
Glucose Monitoring Device			
CGM	3	37.5 %	
Glucose meter	1	12.5 %	
Both (CGM and glucose meter)	4	50 %	

***Caregiver Characteristics (n = 8)***			
Gender			
Female	7	87.5 %	
Male	1	12.5 %	
Age(yrs)	*M =* 45.63	*SD =* 3.81	42–51
Race			
White	7	87.5 %	
Other	1	12.5 %	
Household income (*n* = 11)	*Median* = 158K	*SD* = 97.9K	13,000–300,000
Highest level of caregiver education			
High school degree or GED	1	12.5 %	
Some college, no degree	2	25.0 %	
Associates degree	1	12.5 %	
Bachelor's degree	1	12.5 %	
Master's degree	3	37.5 %	
Employment status			
Full-time	6	75.0 %	
Part-time	1	12.5 %	
Unemployed	1	12.5 %	

### 3.2 Satisfaction with the proposed program

Most participants—both adolescents and caregivers—expressed positive impressions of the program overall. However, one teen participant stated that the program sounded too much like another medical appointment, and one caregiver expressed concern that the program was too focused on weight. Most participants were enthusiastic about the proposed program, stating that it would fill a need in the community and even asking how they could sign up for the first session. For example, one adolescent (13y, Female) stated *“I think like it will help a lot of people … It could make, like, a big change.”* A caregiver (42y, Female) stated *“I think that sounds awesome and I think more kids need that sort of thing. You know ‘cause they are not alone – it's more a support system and educational at the same time.”*

**Mental Health Sessions.** Out of all the proposed topics (e.g., nutrition, physical activity, goal setting, mental health skill building), the mental health-related topics were of particular interest to most participants. Participants noted that mental health topics related to body image and diabetes burnout are of specific importance to youth with T1D. For example, one teen (15y, Female) shared *“I really love the emphasis on mental health. I really love the emphasis on like, a positive image of yourself … I know that if you make those changes to your body, it's not gonna help. You need to make those changes inside*.*”* Additionally, participants identified that there was a lack of focus on the integration of physical and mental health in existing interventions for adolescents with T1D. A caregiver (42y, Female) said *“I feel like these are things that the normal, whatever, diabetic person doesn't even think of … Yeah like diabetes burnout not everybody even knows what that is … So absolutely I think that is awesome.”* Overall, participant feedback highlighted the need for mental health topics to be well-integrated into IHBLT.

**Nutrition Education.** Similarly to feedback on mental health, participants had positive feedback on the planned nutrition education. Participants liked the simplicity of how the information was presented, specific recommendations, and individualized support. Participants were particularly interested in looking at patterns between blood glucose levels and nutrition. One caregiver (42y, Male) summarized this by sharing: *“Even when you see the endocrinologist today, there's no, it's the quick check of the blood sugars, but I'm just saying how it relates to what you're eating … it's huge. But I've never really seen someone take the time to sit down and walk through that.”* Similarly, a teen (15y, Female) stated *“I also love how when you're tracking the blood sugar, you find patterns. So you know how, like certain foods affect your blood sugar. And I feel like if you were in the program you'd be more apt to like, look for those patterns and learn how to control your diabetes better*.*”* A few caregivers stated that the nutrition review included in this program, sometime after initial diagnosis, would be useful to adolescents and their caregivers. They recalled being told by providers to not change their child's diet at diagnosis and/or to be extra cautious about causing eating disorders, given the increased risk in this population. Caregivers described not having any information on how to best support their teens' healthy eating; and both caregivers and teens expressed anxiety related to how to eat well.

**Adolescents Unlikely to Complete Self-Monitoring.** In contrast to the overall satisfaction with intervention components, many participants, especially caregivers, explained that it is unlikely teens will complete 100 % of the self-monitoring components (i.e., food intake and blood glucose tracking) of the proposed program. An adolescent (15y, Male) responded to the explanation of food self-monitoring with the following: *“Um ….I'd probably forget sometimes. But I think that's just part of it … but I definitely think a lot of people wouldn't.”* On the other hand, another teen (16y, Female) said “*Honestly yeah, if I was in the program, I would do it. …”* Participants expressed slightly more hope that teens would engage in self-monitoring if they were incentivized. For example, one caregiver (49y, Female) stated *“It would take commitment, like severe commitment to do that … I think the incentive is good. Plus if they commit to the program they have to want to make a change for themselves.”*

### 3.3 Program ctructure preferences

Beyond general perceptions of the program, participants were also asked to provide feedback on specific aspects of the program, including program length and inclusion of caregivers.

**Length of Program.** While participants had varied opinions, the majority agreed that the planned 16-week intervention seemed too intensive at first. However, once learning about all the topics included, participants were more amenable to the planned 16-week length. A few specifically suggested 10–12 sessions would be more acceptable. A teen (13y, Male) stated “*It seems kind of long …”* and another (13y, Female) said, *“… I think maybe a little shorter like 12 weeks, that would be good.”* Some participants suggested combining topics such that the content could remain in a shorter program length. Alternatively, one caregiver (42y, Female) thought that the program length might be beneficial by saying, *“Well so 16 weeks is a long time, but it could be helpful and it kind of teaches them to stick with something. It's not like I'll be done in 12 weeks and then that's it. It kind of sees them through a long period of time and make them commit to it, and hopefully they learn, and they grow from it.”*

**Number of Caregiver Sessions.** Most participants—both teens and caregivers—agreed that the planned four caregiver-included sessions were the appropriate amount. However, a few teens thought there should be fewer sessions involving caregivers. One teen (13y, Female) stated *“Yeah, I think like every four weeks is good, I think that if they are in here with the kids all the time – it would push the kids away from wanting to talk to one another.”* A caregiver (42, Female) agreed by stating *“I think it's good cause it is including the parents but it's also giving the kids their own time.”* One caregiver (50y, Female) suggested that younger teens may want more parental involvement, and one teen (14y, Female) stated that having caregivers present for the first session might put teens at ease who have some social anxiety. A few caregivers mentioned wanting more caregiver-specific peer support, as this caregiver (44y, Female) suggests *“… maybe while the kids are meeting give the parents the option, if they want, to … have a little parent get together … And there's a lot of emotion and guilt and feeling bad that comes with that – so I definitely think that could be helpful.”*

### 3.4 Suggested program adaptations

As demonstrated with prior themes, participants had positive perceptions of the program overall, yet participants did suggest specific adaptations to the proposed program. Specifically, this centered around framing key topics (including weight management) and additions to the program to complement the proposed intervention topics.

**Framing the Program.** Some participants, mostly caregivers, reported being concerned about discussing weight, given the potential distress for adolescents when discussing these topics. For example, one caregiver (44y, Female) shared, *“I think the weight part of it is really important … it is such a sensitive issue, especially for teenage girls … You know … and … just healthy eating and exercise … I feel like I have to walk on eggshells around it.”* Though caregivers were open to having a safe and professional space for their teens to discuss health behaviors and weight, they also expressed protection for their kids' potential uncomfortable emotions. A few caregivers became emotional during the interviews when discussing their fears related to weight, eating, and/or diabetes distress. One caregiver (49y, Female) said *“… this assumption that the more you weigh, the less healthy you are is not something we really want to be teaching people who have a little less control over what they weigh … I don't think we should be giving kids with a chronic condition a complex about their weight.”*

Along the same lines, other caregivers expressed concerns regarding specific wording proposed such as “feeling different” and “fear of hypoglycemia.” For example, a caregiver (50y, Female) said *“I don't like the word ‘fear of hypoglycemia.’ Like, like she's never said she had a fear of it. but I don't want her to think there's a reason to have a fear of it*.*”* Other participants, teens included, suggested slight adjustments to the way topics were presented such that teens felt more in control. For example, one teen (14y, Female) suggested the program leaders use an objective, non-judgmental tone when talking with teens about their health patterns.

**Additional Topics.** Adolescents and caregivers suggested adding a few other topics to the program. Although there was no consensus among participants in terms of program additions, adolescents suggested including: (1) wearable devices and body image, (2) fear of hypoglycemia and sleep, (3) carb counting education, and (4) representation of diabetes in popular media. Caregivers suggested the following topics: (1) drugs and alcohol, (2) dangers of driving with hypoglycemia, (3) how to best disclose diabetes to a coach/teacher, and (4) managing diabetes-related bullying in school.

## 4 Discussion

There is a critical need for the development of programs specific to the treatment of pediatric obesity among those with T1D [3,4]. This study collected input from adolescents with T1D and with overweight/obesity and their caregivers regarding an IHBLT for youth with T1D and with overweight/obesity. Participants indicated enthusiasm for the proposed program topics and planned activities. Their constructive feedback provided many opportunities to tailor the initially adapted program to their specific needs, with a goal of increasing the likelihood of program's acceptability and feasibility.

Participants discussed T1D management and mental health components more often than weight management components. It is unclear from the data exactly why this occurred; one possibility is that participants felt more comfortable with the former topics. Another possibility is that weight management is less important to them compared to mental wellness and T1D management. Some caregivers were distressed discussing their concerns regarding eating disorders or adolescents' distress about weight status, given the high prevalence of disordered eating among this population [[14], [15], [16]]. Yet, adolescent feedback was consistent with previous work, indicating comfort and interest in discussing weight [19]. An IHBLT can provide a safe space for teens and skills for caregivers to have these conversations. It may also be that a program that less explicitly uses the words “weight management” but retains a focus on promoting health behavior that supports healthy weight (e.g., balanced diet, increasing physical activity) may balance parent and patient preferences while aligning with previous evidence and theory. Prior work examining barriers to traditional IHBLTs suggest that an emphasis on weight change can be a barrier to participation due to the high societal stigma [25]. There is limited evidence on the efficacy of weight-neutral approaches to weight management, particularly for adolescents, though the literature supports participants acceptability and feasibility of them [26,27]. Participants also stated additional topics related to T1D health and wellness that they would like to see incorporated into the program. These topics included a review of diabetes management recommendations more specific to adolescents and young adults, as well as other mental-health-related topics (e.g., fear of hypoglycemia and bullying/teasing).

Peer support was a strength identified within the adapted IHBLT. Participants discussed how the opportunity to meet other teens with T1D would attract them to the program. Teens and caregivers acknowledged minimal caregiver involvement is preferred, as caregivers can dominate conversations. Given that family support is linked to positive physical and mental health outcomes among youth with T1D [28,29], it may be beneficial to provide caregivers with skill-building to support their teens in one setting, while teens have a separate space to share openly with their peers in a group moderated by a health professional.

Some participants noted the 16-week program length may be too intensive and that they would be unlikely to complete daily self-monitoring activities. National guidelines (USPSTF, AAP) state that IHBLTs should include *at least* 26 h of face-to-face treatment over 3–12 months, and include that self-monitoring is one of the strongest predictors of weight-loss success [8,9]. These findings parallel prior work, in which providers and patients have voiced skepticism and practical barriers regarding the feasibility of completing the suggested intensity of a standard IHBLT [30,31]. While time-intensive IHBLTs may be more effective, committing to such a program may be unrealistic for many, thereby inhibiting access. Thus, a future 12-session IHBLT would be more acceptable to this population. It will be important for future researchers to test the effectiveness of both a full-dose and briefer version of an IHBLT for this population. Previous work has also identified logistical barriers to traditional adolescent IHBLTs such as transportation, caregiver work, and family scheduling [25]. A future T1D-specific IHBLT should also consider strategies that mitigate these barriers, such as virtual programming, childcare support, and/or transportation assistance. Similarly, while self-monitoring is associated with positive behavior change [9], results indicate barriers to self-monitoring. Recent technological advances allow for more passive self-monitoring (e.g., CGMs, bite counts). However, passive data collection may inhibit one's ability to build insight and motivation for behavioral change. Given that most adolescents in this study used CGMs, future IHBLTs should consider strategies to balance less burdensome self-monitoring with strategies that help teens to develop insight. Youth with T1D need support in recognizing individual patterns between their eating and activity and their blood glucose trajectories. Future interventions should consider strategies such as engaging reminders, incentives, and gamification strategies to increase adolescents' motivation for self-monitoring.

## 5 Limitations

Including the target population in the development of a clinical intervention is a major strength of this study. This strategy offers a practical method for building collaboration with the target patient community as well as increases the likelihood of acceptance by the target population. However, this study is limited in a few ways. First, the findings are preliminary and hypothesis generating; thus the results should be interpreted with caution. The sample is relatively small and homogeneous with regard to race and gender. This limits the generalizability of the results to the entire adolescent T1D population. Though there was interest in the study, only 20 % of those interested completed the study. Nearly half of the people who expressed interest in the study could not be reached to confirm eligibility and/or schedule study sessions. As participants were only eligible if they spoke English fluently, our findings are limited, and future work is needed to explore this work in families with a primary language other than English. Another limitation is the potential bias in those who agreed to participate in the study. The study was advertised as a one-time interview for adolescents with T1D and their caregivers regarding the development of a new healthy lifestyle program. It is possible that families who are more personally interested in healthy lifestyles sought out participation in this study. The results, therefore, may be missing important input from adolescents and caregivers who are less interested and/or motivated to participate in an IHBLT. Hearing from the voices of those participants who are harder to reach, such as participants who are less interested in behavioral programming and samples with higher burden, such as families experiencing multiple social determinants of health, might elucidate other necessary refinements and strategies to increase reach.

## 6 Conclusion

The results offer one step towards informing future researchers in the design and implementation of healthy lifestyle and diabetes-specific programs for adolescents with T1D. Iterative change to the adapted IHBLT for T1D should consider: (1) reducing the program length to maximize engagement, (2) including optional or more passive self-monitoring activities, (3) primarily focusing on T1D management and mental health topics as opposed to weight and shape, (4) additional parent/caregiver support groups, and (5) inclusion of topics related to eating disorders, diabetes distress, and healthy weight management for caregivers of adolescents with T1D. Future research is needed to examine how suggestions offered by participants in the current study will impact participation and health outcomes in a future IHBLT program specific to youth with T1D. The research team is currently examining the acceptability and feasibility of an IHBLT for adolescents with T1D based upon the results of this study.

## Declaration of competing interest

The authors declare the following financial interests/personal relationships which may be considered as potential competing interests: Jennifer Warnick reports financial support was provided by 10.13039/100000062National Institute of Diabetes and Digestive and Kidney Diseases. Katherine Darling reports financial support was provided by 10.13039/100000062National Institute of Diabetes and Digestive and Kidney Diseases. KayLoni Olson reports financial support was provided by 10.13039/100000062National Institute of Diabetes and Digestive and Kidney Diseases. If there are other authors, they declare that they have no known competing financial interests or personal relationships that could have appeared to influence the work reported in this paper.
